# A Constructivist Grounded Theory Study on Mental Health Recovery from a Lived Experience Perspective in Singapore

**DOI:** 10.1007/s10597-023-01184-0

**Published:** 2023-09-25

**Authors:** Jonathan Han Loong Kuek, Toby Raeburn, Melissa Yan Zhi Chow, Timothy Wand

**Affiliations:** 1 Susan Wakil School of Nursing and Midwifery, Sydney Nursing School, Faculty of Medicine and Health, The University of Sydney, New South Wales, 2006 Australia; 2 Faculty of Nursing and Midwifery, Health Sciences & Physiotherapy, The University of Notre Dame, New South Wales, 2010 Australia; 3 School of Social Sciences, Nanyang Technological University, Singapore, Singapore

**Keywords:** Recovery, Mental illness, Mental disorder, Singapore, Asia

## Abstract

More contemporary personal recovery conceptualisation of mental health recovery emphasize the need to consider the perspectives of people who experience mental ill-health. Most lived experience research has been done in Western cultures with relatively few studies in Asian ones, creating a gap that needs to be addressed due to differences in cultural worldviews. This study explores the notion of recovery from the lens of people experiencing mental health challenges in Singapore. We adopted a constructivist grounded theory perspective to evaluate qualitative data from 21 participants. The core category which best represented what recovery meant was “reconciling and living with experiences of mental ill-health”. Our findings suggest that a variety of societal aspects greatly influence perceptions of mental health recovery in Singapore, as participants often shared their desire to live a meaningful life within society but could only do so if they found a way to manage their symptoms more effectively.

More than 1.1 billion have experiences of mental ill-health, making mental healthcare an important priority for healthcare systems (Dattani et al., 2021). In the past, recovery from mental illnesses was often defined as the reduction of symptoms and return to daily life and mental health professionals were viewed as experts on how this could occur (Jacob, 2015; McCabe et al., 2018; Stein et al., 2010). While suchviews are still held by many, a more contemporary approach promoted by the personal recovery movement, has gained traction and popularity since it was first put forth in the 1990s (Anthony, 1993; Deegan, 1988; Slade et al., 2012).

From a personal recovery perspective, people who engage with mental health services are viewed as experts of their experience and mental health services seek to to be include them in all aspects of care. Consequently, instead of being directive and prescriptive, mental health professionals take on a more supportive role which seeks to better understand the desires of the people they are supporting to derive goals that are meaningful to the individual (Amering, 2012; Leamy et al., 2011). Due to this paradigm shift, there has also been growing interest in lived experience research and involving people with experiences of mental ill-health to better appreciate their perspectives (Cleary et al., 2013; Gillard et al., 2015; Kidd et al., 2015; Lloyd-Evans et al., 2014; McKenna et al., 2014; Slade et al., 2014, 2015; Slade & Longden, 2015; Wyder & Bland, 2014).

However, many early conceptualizations of personal recovery have emerged from western oriented cultures and are charecterised by a heavy emphasis on individual-centric aspects of mental health recovery (Jacob, 2015; Jacobson & Farah, 2012; Leamy et al., 2011). One of the most often cited definitions of personal recovery, by William Anthony, suggests recovery has more to do with the person as it is a “deeply personal, unique process of changing one’s attitudes, values, feelings, goals, skills, and/or roles. It is a way of living a satisfying, hopeful, and contributing life even with the limitations caused by the illness” than predominantly based on his/her environment and social influences (Anthony, 1993). While this maybe true to some extent, as mental health recovery is inherently tied to the experiences of an individual, in many Asian societies, socioenvironmental influences play a powerful role in shaping the lives of people cannot be discounted (Jacobson & Farah, 2012; McCabe et al., 2018; Waller et al., 2019).

For example, a study in Indonesia which peoples experiences of recovery from psychosis, highlighted how it was viewed as the ability to exist within various physical communal spaces and being able to slowly progress to such a point was the journey they needed to take in order to recovery (Subandi, 2015). Another study in Thailand, which focused on women who were struggling with alcohol addiction showed that they were hindered and supported in their recovery due to various gendered and social norms whichinfluenced the expectations of others which in turn impacted the way they viewed their recovery journey (Hanpatchaiyakul et al., 2017).

Indeed, an emerging body of personal recovery literature from various Asian cultures supports the notion that more culturally appropriate and theory-building approaches need to be adopted when applying principles of the movement in societies where lived experience studies are sparse (Bingham & Kelley, 2022; Efendi et al., 2022; Lee et al., 2022; Linde et al., 2022; Suryani et al., 2022; Tse et al., 2021; Tsoi et al., 2022; Yoder et al., 2021; Yu et al., 2021; Yung et al., 2021). This need results from the complex interplay between societies and the individual in relation to how recovery is perceived. Singapore is a highly diverse and multi-cultural nation consisting of four main racial groups (Chinese, Malay, Indian, and Others), suggesting that much like the other studies in Asia briefly discussed above, significant differences in how mental health recovery is viewed can be expected and are worth exploring. Hence, we sought to further add to these efforts by diving into the lived experiences of mental ill-health in the Singapore context and better understanding how they conceptualized the idea of recovery. The aim was to better learn about the meanings attributed to mental health and recovery by people who experienced them in Singapore.

## Method

We adopted a constructivist grounded theory perspective to understand how our participants construed the realities they lived in to better unpack the intricacies of their experiences (Charmaz, 2017a, b). The qualitative data we used in this study came from people with mental health challenges collected as part of a broader project studying mental health recovery in Singapore. Only people from the lived experience population was included in this segment and individuals such as mental health professionals and informal caregivers were not discussed. We adopted the recommendations put forth by the Consolidated Criteria for Reporting Qualitative Research checklist (Tong et al., 2007) while preparing this paper.

### Ethics Statement

Before starting the study, we obtained ethical approval to carry out this mental health recovery project from the Human Research Ethics Committee at the University of Sydney (2019/934). Verbal and written informed consent was also received before any interviews commenced.

### Recruitment

Intentional sampling was used to recruit people with various experiences of mental ill-health to provide better representation within the data collected, congruent with constructivist grounded theory procedures. They were invited to participate in the study via various social media and messaging platforms (LinkedIn, Facebook, Telegram, WhatsApp, and Instagram), where details of the study were shared in informal mental health groups in Singapore, and they needed to sign up for it.

### Interview Process

Online zoom interviews were conducted between May 2021 and November 2021 as the COVID-19 pandemic and movement restriction protocols in Singapore at the time of data collection prevented face-to-face meetings from taking place. The first author conducted all the interviews to ensure consistency, and a semi-structured interview schedule was used during the process. Examples of questions include – “what does mental health recovery mean to you?”, “do you think recovery is possible?”, and “what would successful recovery look like?”. The interviewer also took field notes during the sessions that were later used while reviewing and analysing the data.

### Reflexivity

Upon completion of each interview, the first author (i.e., the main interviewer) engaged in active reflection to identify the possibilities of his biases colouring the session in any way. While complete objectivity may not be possible as researchers unconsciously bring various influences from their life to these sessions, such sessions allowed these potential biases to be reduced (Charmaz, 2017a, b). Additionally, two Australian mental health academics were also included in every step of the study and safeguards were put in place to ensure the impact of biases was mitigated. For example, the interviewer carefully followed the interview guides and only requested elaboration points raised during the session. Furthermore, if external information was brought in, only points raised by other participants were allowed as a way to validate them with new perspectives from different people. Lastly, the two Australian researchers also reviewed the interview sessions to ensure that they were not becoming directive.

### Data Analysis

Interview recordings were first transcribed and uploaded onto a qualitative data management software (i.e., NVivo) for coding. This process was guided by the constructivist grounded theory approach (Charmaz, 2017a, b; Mills et al., 2006) which provided instructions on how coding should be done and considerations that needed to be present while doing so; for example, including the need for flexibility and creativity, or recognition of potential biases and existing theoretical knowledge. Initial codes became the basis of various sub-categories and categories. A single core category was also identified in the process to represent all the data in line with constructivist grounded theory approaches. Discussions functioned on a constant comparison, and consensus-based approach where disagreements were discussed and voted upon before decisions to keep or change them were made. All the data was also constantly compared with new codes, sub-categories, and categories to see how they fit within the broader context of the study. As the process of coding within the constructivist grounded theory framework was an ongoing one, member checking (i.e., sharing with each new participant a brief summary of what has been found thus far) was also done to ensure our preliminary results and sub-categories were developing in a coherent fashion. All authors agreed upon the final results.

## Results

Demographic information of the 21 people who had received diagnoses of mental health conditions who participated in this study can be found in Table 1.

**Table 1:** Demographic of participants

S/N	Age	Race	Religion	Gender	Employment Status	Mental Health Condition	Duration with Condition
PIR0001	24	Indian	Freethinker	Female	Unemployed	GAD^1^/MDD^2^	10 years
PIR0002	21	Malay	Muslim	Female	Student	MDD/BPD^3^	5 years
PIR0003	21	Chinese	Freethinker	Female	Student	PD^4^/MDD	5 years
PIR0004	27	Chinese	Freethinker	Female	Student	MDD/GAD	12 years
PIR0005	24	Chinese	Freethinker	Male	Student	PDD^5^	15 years
PIR0006	23	Chinese	Christian	Female	Working	MDD	6 years
PIR0007	31	Chinese	Christian	Female	Working	MDD	9 years
PIR0008	27	Chinese	Christian	Female	Unemployed	MDD/Social Anxiety	9 years
PIR0009	22	Chinese	Freethinker	Female	Student	MDD/Bulimia	2 years
PIR0010	25	Chinese	Agnostic	Female	Unemployed	MDD/GAD	7 years
PIR0011	22	Chinese	Catholic	Female	Unemployed	MDD/GAD	4 years
PIR0012	23	Caucasian	Freethinker	Female	Working	MDD/GAD/BPD	5 years
PIR0013	23	Chinese Eurasian	Catholic	Female	Unemployed	MDD/GAD/OCD^6^	9 years
PIR0014	25	Chinese	Freethinker	Female	Unemployed	MDD	1 year
PIR0015	45	Chinese	Christian	Female	Working	Schizophrenia	8 years
PIR0016	21	Chinese	Freethinker	Female	Student	MDD/Social Anxiety	2 years
PIR0017	27	Chinese	Buddhist	Female	Unemployed	MDD/GAD/BPD	14 years
PIR0018	51	Chinese	Christian	Male	Working	MDD	24 years
PIR0019	23	Indian	Atheist	Male	Student	GAD	2 years
PIR0020	43	Chinese	Freethinker	Male	Working	BD^7^	20 years
PIR0021	46	Chinese	Christian	Female	Working	Complex Trauma	22 years

^1^GAD – Generalized Anxiety Disorder; ^2^MDD – Major Depressive Disorder; ^3^BPD – Borderline Personality Disorder; ^4^PD – Panic Disorder; ^5^PDD – Persistent Depressive Disorder; ^6^OCD – Obsessive Compulsive Disorder; ^7^BD – Bipolar Disorder

Our analysis revealed the core category explaining how mental health recovery is conceptualized as “reconciling and living with experiences of mental ill-health”. It can be further represented by three categories: “fluidity of mental ill-health and recovery”, “sliding scale of function”, and “moving on and returning to society”.

### Fluidity of Mental Ill-Health and Recovery

When participants shared about their visions of recovery, they often struggled to encapsulate their experiences and attributed this to the inability to quantify experiences of mental ill-health effectively, unlike physical health conditions. This difficulty could be seen through their hesitation to provide a fixed idea of what recovery could look like, and how they were few indicators to lean on in relation to knowing when they could be deemed recovered.

“for physical recovery, we know that when we are injured uh if there is a cut or whatever, upon having proper treatment, over time, will recover and will recover totally and mental health for me even for myself and my own experience is that you don’t even know if you have recovered totally or not and what is total recovery what does it even mean you know” – PIR0018.

“a physical illness, it shows that like it’s gone, there’s some form of, you’re able to measure it or like test it. But for mental, it’s harder, cause even from a third person point of view, like a professional, I think there are limitations to how they diagnose and how they can identify.” – PIR0009.

Some viewed mental ill-health as part of a larger spectrum of normal human experiences and that they were not something that could be gotten rid of. To this group, there seemed to be some attempt at trying to incorporate their experiences into everyday life and by doing so, reconciled with their conditions and symptoms.

“…like no matter how happy go lucky or positive a person can be, there will always be times where they feel down, they will struggle, and there will be down periods, it’s just like the way they cope is like in a healthier manner?” – PIR0009.

“…personally no one will really successfully recover, because I think ultimately mental health is part of your whole health, your physical health, and what not. And whatever life throws at you, you are going to experience different sense of anxiety, different sense of stress and stuff like that…” – PIR0007.

While others viewed these experiences of mental ill-health as something whereby there was no recovery from, and things would never go back to how they used to be, even though they felt it was necessary to do so because of cultural norms. For those who viewed their conditions in this manner, a sense of resignation and defeat could often be felt, but also a willingness to press on in the face of constant setbacks.

“I cannot see myself as recovered yeah because I have lived with it (mental ill health) for so long its just going to live as a part of me is like a scar that is going to live together” – PIR0008.

“I think the Singaporean Asian culture of like if something back happens you triumph over it and like you achieve something so even in your battle with mental health, like you don’t co-exist peacefully with it, but you like achieve and overcome it” – PIR0019.

### Sliding Scale of Function

While participants were unsure about the possibility of recovery, it was still vitally important that they were able to gain greater functionality and find ways to deal more effectively with their various symptoms or situations. Being able to rely less on medication was a popular indicator that they were improving as participants shared about the stigma associated with being on it.

“a certain remark you know in Hokkien like, this person very weird ah, like this morning you never take medicine. And this is a very negative connotation of people having to take medication especially for mental health issues you see” – PIR0018.

“I think maybe medications would be a problem, like I would definitely feel very uncomfortable talking about that if I were doing it again that’s why I don’t want to do it also because I would find it very hard to share that I’m on medications. People can be very insensitive sometimes and all that kind of things…” – PIR0004.

Additionally, they expressed a desire to be able to cope better and manage various challenges and stressors that were thrown their way as being vital to gaining more autonomy over their mental health so that experiences of mental ill-health would not affect their lives as much.

“I would like to have better control and um like over my episodes and over how I allow them to let my productivity be affected that sort of thing, and develop good coping skills that when emotionally intense moments come, I can deal with them so that they aren’t so catastrophic.” – PIR0006.

“I guess I would say just maybe having a bit more ownership of myself so that I don’t feel so dependent on external things even if there is a time now when I’m doing worse or you know might be seen as a relapse or specific symptom popping up again, I guess being in recovery would mean I have more tools and ability and control over it” – PIR0012.

It was also shared that participants expected many ups and downs throughout what they described to be an ongoing process of trying to improve their function and regain a greater sense of control over their mental health.

“there’s no concrete line in the sense where okay you’re done, you’re healed. You’re perfectly healthy and there’s no more to be done um because I think life keeps happening right and there’s always going to be ups and downs and it’s going to take constant work to ensure you are maintaining this certain level of wellness or adjustment in life. And you can be healthy but then slide back if you stop giving it the kind of attention, you stop giving it a kind of priority.” – PIR0014.

“it’s just a journey for me. Like strangely enough when I look back, it’s like it morphs my symptoms. Like it started off with panic attacks then it changes to binge eating and food restrictions so like there were just different symptoms where I go through in this recovery or life. And every time something new hits, you will learn how to deal with them.” – PIR0003.

### Moving on and Returning to Society

Ultimately, what participants wanted was to be able to find a way to move forward in their lives and return to living within society in a productive manner. In some cases, it would be so they could fulfill the roles they were already playing or wanted to play in the future.

“…successful recovery to me would look like that I’m able to function well everyday in society, at work, at home, leading a normal life, and contribute my part, and I can help others, and perform my work well at home, and can be a good husband, good father, responsible you know, a child to my parents, I make a contribution to society, so that would be to me a very successful life and so this is what I see that I could perform that right now and to continue to do so” – PIR0018.

“…go to university, finish degree, start family. You know that kind of thing? feel like a normal person. Not having so many anxiety attacks, depressive episodes, not thinking suicidal thoughts, and being back to how I was. Like I want to live life when I was 13 and below, like having that capacity and potential and that functioning level will be recovery” – PIR0013.

For others, this return to society was necessary so that the people supporting them could move on with their lives as well and did not need to worry about them.

“I feel that as much as I am the one currently facing this particular condition, the people around me are affected, in a way that they have to be a lot more understanding and they have to have a lot more empathy…” – PIR0001.

“…it wasn’t easy to get out of the hospital to be honest… and I didn’t want to have to go through that again. And then also for my mum I don’t want her to go through that again, it really motivates me to move forward.” – PIR0004.

Moving forward also meant being able to feel more comfortable in their social worlds, free from the internalized stigma that often accompanied their mental health challenges, a state where they could feel able to live as they desired.

“it’s like you no longer feel the need and shame as much when you make a mistake so it’s about how you feel and how you how you interact with the world. The way you are living in the way you desire to live” – PIR0011.

“I know that for some reason I don’t think I deserve to be called ill in the way that like…I don’t think I can live up to the criteria. Ok not that I feel embarrassed by being ill but more like weak. Don’t sympathize with me because you are just making me be more invalidating of myself.” – PIR0010.

## Discussion

Our findings suggest that participants viewed recovery as a reconciliation process and learning how to live with their experience of mental ill-health. Additionally, there was an overarching sense of resignation during their sharing, especially when discussing the possibility of recovery based on their definitions. These feelings were often reflected in their difficulty in concretely defining recovery even when given free rein, as seen in the first category, which captured the fluidity of mental ill-health and recovery. This inability to easily articulalte their thoughts about mental ill-health, and consequently, the idea of recovery, could perhaps be explained by the generally low levels of mental health literacy in Singapore (Tonsing, 2018). Coupled with the high levels of stigma in the country, interest in better understanding experiences of mental ill-health is often low, resulting in a cycle of low literacy unless there is a need to learn about them (Subramaniam et al., 2020; Yuan et al., 2016). Consequently, while this was not a formal theme, participants frequently shared that they had never considered these issues before participating in this study.

Furthermore, it could also speak to an internal conflict between the culture they were brought up in, which emphasizes the need to overcome such challenges, the chronicity of mental health challenges, and not having other definitions of recovery to rely on beyond how physical health conditions are viewed. These findings echo those of other studies in Asia, whereby recovery was often found to be challenging to define and strongly influenced by the cultural setting of the society (Della et al., 2021; Hanpatchaiyakul et al., 2017; Lemelson & Tucker, 2017; Subandi, 2015; Subandi et al., 2021; Yoder et al., 2021). However, it is worth noting that some participants did share the need to view mental ill-health as part of broader human experiences and that it was a part of them, a perspective that is congruent with how personal recovery is often discussed (Anthony, 1993; Jacob, 2015; Slade et al., 2012). Perhaps introducing different concepts to people unsure of their definitions could give them more options to choose from, which may reduce the ambivalence experienced.

The desire for greater autonomy was also identified as participants sought to regain control over their lives following the onset of mental ill-health. Specifically, one key aspect was being able to cope with their symptoms more effectively, and they often used medication as a yardstick for improvement. From their view, there was a certain level of stigma toward psychotropic medication in Singapore, which is supported to a certain extent by a study that showed that people in the country turn to complementary and alternative sources of help-seeking before medical pathways for mental ill-health four times more than people without a mental health challenge (Seet et al., 2020). Additionally, other studies in Singapore and various Asian contexts suggest that there could be cultural perspectives toward these experiences, such as people being weak or possessed and taking medication would be a public show being someone who was part of people who were viewed that way, further exacerbating the fear of medication (Ran et al., 2021; Subandi et al., 2021; Suryani et al., 2022; Tan et al., 2020; Tasijawa et al., 2021; Yoder et al., 2021).

Many believed this could be done by learning more skills and coping techniques to gradually gain greater autonomy over their experiences of mental ill-health and medication could be tapered off more effectively. Such an approach has been supported by literature that suggests it is possible (Lee et al., 2021) but remains a contentious point as there could be resistance from people who believe that medication is essential to a holistic recovery (Druss et al., 2018; Jessell & Stanhope, 2021). Additionally, recent evidence emphasising the lack of biological etiology for common experiences of mental ill-health, such as depression, and their implications on the utility of medication further complicate these discussions (Moncrieff et al., 2022). Taken together, perhaps this demonstrates the individualized nature of the recovery process where everyone needs to identify the most appropriate strategies while factoring in their social environments.

The inclusion of social factors in their consideration was crucial as participants shared their desires to reach a state whereby they could play the roles they believed they were meant to, especially concerning their direct social environments. This echoes various studies in Singapore and Asia that have similarly identified family and society as being strong motivators for people wanting to get better (Lai et al., 2021; Lam et al., 2021; Suryani et al., 2022; Yuen et al., 2019). In particular, this desire to contribute back to society or to be viewed as a normal part of it seem to be salient and uniquely Asian-oriented values, and there appears to be a difference in the way relationships between self, family, and society are conceptualized from countries which may be a bit more individualistic. However, it is also important to note that western cultures, where most individualistic countries stem from, are not homogenous, and there could be significant cultural differences that need to be considered (Linde et al., 2022; Nwokoroku et al., 2022). Hence, these cultural and societal nuances need to be adequately accounted for where lived experience research is concerned.

Moreover, stigma can play a huge role in preventing them from playing more active parts in society, as participants often shared how they did not want to be viewed as weak or feel the shame associated with experiences of mental ill-health. These negative feelings were worsened when they started implicating their families by association and could explain why many thought they had to find a way to live with their experiencesconditions more effectively and in a societally accepted manner. Hence, efforts to reduce stigma and improve the mental health literacy in a culturally appropriate manner need to occur so experiences of mental ill-health can be understood by more people. Doing so requires an intentional effort to share easily understood information and incorporates their existing understandings (Subandi et al., 2021; Yoder et al., 2021).

### Limitations

Its size and scope of inquiry limited our study, but the lack of generalizability was mitigated to a certain extent by our ability to dive deeper into the nuances underlying our findings. However, future studies will still need to carefully extrapolate and quantify what we have found here to test for broader application across a more comprehensive range of people experiencing mental ill-health. Additionally, our sample was predominantly comprised of people experiencing depression and anxiety-like symptoms. Although these are two of the more common experiences of mental ill-health in Singapore, they could also reduce the utility of our findings for people with more debilitating mental health challenges, which future studies will need to explore as well. Furthermore, as we were focusing on understanding broadly how recovery was viewed from the context of people experiencing various mental health issues, we did not dive deep into the experiences of any specific mental health condition. Hence, comparisons between our results and studies that focused on single conditions could not be done. Nevertheless, our results still provide a high level overview of how the experience of mental ill-health and recovery in Singapore could be perceived.

## Conclusion

Our study provided helpful insight into the perspectives of people experiencing mental ill-health, specifically in understanding how they viewed the idea of recovery. We found a bleak picture and sense of resignation in their situations while also holding a sense of hope that things may become better eventually. Societal influences played a substantial role in shaping these views, and participants mainly wanted to return to society and live their desired lives. Future research needs to better understand how and why these influences have such a salient impact on the views shared. In doing so, we can perhaps better understand their perspectives and ideas around recovery more completely and allow for better interventions to be developed to support people who experience mental ill-health more effectively.

## References

[CR1] Amering M (2012). Recovery, science and human rights. Epidemiol Psychiatr Sci.

[CR2] Anthony WA (1993). Recovery from mental illness: The guiding vision of the mental health service system in the 1990s. Psychosocial Rehabilitation Journal.

[CR3] Bingham, D., & Kelley, A. (2022). Rethinking recovery: A qualitative study of american indian perspectives on peer recovery support. *Journal of Ethnicity in Substance Abuse*, 1–14. 10.1080/15332640.2022.2082620. 10.1080/15332640.2022.2082620 35678280

[CR4] Charmaz, K. (2017a). Constructivist grounded theory. *The Journal of Positive Psychology*.

[CR5] Charmaz K (2017). The power of constructivist grounded theory for critical inquiry. Qualitative Inquiry.

[CR6] Cleary M, Horsfall J, O’Hara-Aarons M, Hunt GE (2013). Mental health nurses’ views of recovery within an acute setting. International Journal of Mental Health Nursing.

[CR7] Dattani, S., Ritchie, H., & Roser, M. (2021). *Mental Health. Our World in Data* https://ourworldindata.org/mental-health.

[CR8] Deegan PE (1988). Recovery: The lived experience of rehabilitation. Psychosocial Rehabilitation Journal.

[CR9] Della CD, Teo DCL, Agiananda F, Nimnuan C (2021). Culturally informed psychotherapy in asian consultation-liaison psychiatry. Asia Pac Psychiatry.

[CR10] Druss BG, Singh M, von Esenwein SA, Glick GE, Tapscott S, Tucker SJ, Lally CA, Sterling EW (2018). Peer-led self-management of General Medical Conditions for patients with Serious Mental Illnesses: A Randomized Trial. Psychiatric Services (Washington, D. C.).

[CR11] Efendi F, Aurizki GE, Yusuf A, McKenna L (2022). Not shifting, but sharing: Stakeholders’ perspectives on mental health task-shifting in Indonesia. BMC Nurs.

[CR12] Gillard S, Holley J, Gibson S, Larsen J, Lucock M, Oborn E, Rinaldi M, Stamou E (2015). Introducing new peer worker roles into Mental Health Services in England: Comparative case Study Research across a range of Organisational Contexts. Administration and Policy in Mental Health.

[CR13] Hanpatchaiyakul K, Eriksson H, Kijsomporn J, Ostlund G (2017). Lived experience of Thai Women with Alcohol Addiction. Asian Nurs Res (Korean Soc Nurs Sci).

[CR14] Jacob, K. (2015). Recovery model of mental illness: A complementary approach to psychiatric care. 10.4103/0253-7176.155605 PMC4418239 25969592

[CR15] Jacobson N, Farah D (2012). Recovery through the Lens of Cultural Diversity. Psychiatric Rehabilitation Journal.

[CR16] Jessell L, Stanhope V (2021). How do you try to have anyone comply or at least be pliable with you if that person’s not even medicated?: Perspectives on the use of psychiatric medication within recovery-oriented practice. Psychiatric Rehabilitation Journal.

[CR17] Kidd S, Kenny A, McKinstry C (2015). Exploring the meaning of recovery-oriented care: An action-research study. International Journal of Mental Health Nursing.

[CR18] Lai DWL, Chan KC, Daoust GD, Xie XJ (2021). Hopes and wishes of clients with mentally illness in Hong Kong. Community Ment Health J.

[CR19] Lam, J. S. H., Links, P. S., Eynan, R., Shera, W., Tsang, A. K. T., Law, S., Fung, W. L. A., Zhang, X., Liu, P., & Zaheer, J. (2021). I thought that I had to be alive to repay my parents: Filial piety as a risk and protective factor for suicidal behavior in a qualitative study of chinese women. *Transcultural Psychiatry*, 13634615211059708. 10.1177/13634615211059708. 10.1177/13634615211059708 PMC8859686 34928737

[CR20] Leamy M, Bird V, Le Boutillier C, Williams J, Slade M (2011). Conceptual framework for personal recovery in mental health: Systematic review and narrative synthesis. British Journal of Psychiatry.

[CR21] Lee MY, Eads R, Yates N, Liu C (2021). Lived experiences of a sustained Mental Health recovery process without Ongoing Medication Use. Community Ment Health J.

[CR22] Lee, Y. Y., Seet, V., Chua, Y. C., Verma, S. K., & Subramaniam, M. (2022). Growth in the Aftermath of Psychosis: Characterizing Post-traumatic Growth in Persons With First Episode Psychosis in Singapore. *Frontiers in Psychiatry, 12*. 10.3389/fpsyt.2021.784569. 10.3389/fpsyt.2021.784569 PMC8825783 35153855

[CR23] Lemelson R, Tucker A (2017). The bird dancer and the warrior king: Divergent lived experiences of Tourette syndrome in Bali. Transcultural Psychiatry.

[CR24] Linde J, Schmid MT, Ruud T, Skar-Froding R, Biringer E (2022). Social factors and recovery: A longitudinal study of patients with psychosis in Mental Health Services. Community Ment Health J.

[CR25] Lloyd-Evans B, Mayo-Wilson E, Harrison B, Istead H, Brown E, Pilling S, Johnson S, Kendall T (2014). A systematic review and meta-analysis of randomised controlled trials of peer support for people with severe mental illness. BMC Psychiatry.

[CR26] McCabe R, Whittington R, Cramond L, Perkins E (2018). Contested understandings of recovery in mental health. Journal of Mental Health (Abingdon, England).

[CR27] McKenna B, Furness T, Dhital D, Ennis G, Houghton J, Lupson C, Toomey N (2014). Recovery-oriented care in acute inpatient mental health settings: An exploratory study. Issues in Mental Health Nursing.

[CR28] Mills J, Bonner A, Francis K (2006). The development of constructivist grounded theory. International Journal of Qualitative Methods.

[CR29] Moncrieff J, Cooper RE, Stockmann T, Amendola S, Hengartner MP, Horowitz MA (2022). The serotonin theory of depression: A systematic umbrella review of the evidence. Molecular Psychiatry.

[CR30] Nwokoroku, S. C., Neil, B., Dlamini, C., & Osuchukwu, V. C. (2022). A systematic review of the role of culture in the mental health service utilisation among ethnic minority groups in the United Kingdom. *Global Mental Health*, 1–10. 10.1017/gmh.2022.2. 10.1017/gmh.2022.2 PMC9806997 36618728

[CR31] Ran MS, Hall BJ, Su TT, Prawira B, Breth-Petersen M, Li XH, Zhang TM (2021). Stigma of mental illness and cultural factors in Pacific Rim region: A systematic review. BMC Psychiatry.

[CR32] Seet V, Abdin E, Vaingankar JA, Shahwan S, Chang S, Lee B, Chong SA, Subramaniam M (2020). The use of complementary and alternative medicine in a multi-ethnic asian population: Results from the 2016 Singapore Mental Health Study. BMC Complement Med Ther.

[CR35] Slade M, Longden E (2015). Empirical evidence about recovery and mental health. BMC Psychiatry.

[CR36] Slade M, Williams J, Bird V, Leamy M (2012). Recovery grows up. Journal of Mental Health (Abingdon, England).

[CR33] Slade M, Amering M, Farkas M, Hamilton B, O’Hagan M, Panther G, Perkins R, Shepherd G, Tse S, Whitley R (2014). Uses and abuses of recovery: Implementing recovery-oriented practices in mental health systems. World Psychiatry.

[CR34] Slade M, Bird V, Clarke E, Le Boutillier C, McCrone P, Macpherson R, Pesola F, Wallace G, Williams J, Leamy M (2015). Supporting recovery in patients with psychosis through care by community-based adult mental health teams (REFOCUS): A multisite, cluster, randomised, controlled trial. The Lancet Psychiatry.

[CR37] Stein DJ, Phillips KA, Bolton D, Fulford KW, Sadler JZ, Kendler KS (2010). What is a mental/psychiatric disorder? From DSM-IV to DSM-V. Psychological Medicine.

[CR38] Subandi MA (2015). Bangkit: The processes of recovery from First Episode Psychosis in Java. Culture, Medicine and Psychiatry.

[CR39] Subandi MA, Praptomojati A, Marchira CR, DelVecchio Good MJ, Good BJ (2021). Cultural explanations of psychotic illness and care-seeking of family caregivers in Java, Indonesia. Transcultural Psychiatry.

[CR40] Subramaniam M, Abdin E, Vaingankar JA, Shafie S, Chua HC, Tan WM, Tan KB, Verma S, Heng D, Chong SA (2020). Minding the treatment gap: Results of the Singapore Mental Health Study. Social Psychiatry and Psychiatric Epidemiology.

[CR41] Suryani S, Hidayah N, Sutini T, Al-Kofahy L (2022). The indonesian survivors’ perspective about recovery from Schizophrenia: An exploratory study. Jurnal Keperawatan Padjadjaran.

[CR42] Tan GTH, Shahwan S, Goh CMJ, Ong WJ, Wei KC, Verma SK, Chong SA, Subramaniam M (2020). Mental illness stigma’s reasons and determinants (MISReaD) among Singapore’s lay public - a qualitative inquiry. BMC Psychiatry.

[CR43] Tasijawa FA, Suryani S, Sutini T, Maelissa SR (2021). Recovery from ‘schizophrenia’: Perspectives of mental health nurses in the Eastern island of Indonesia. Belitung Nursing Journal.

[CR44] Tong A, Sainsbury P, Craig J (2007). Consolidated criteria for reporting qualitative research (COREQ): A 32-item checklist for interviews and focus groups. International Journal for Quality in Health care.

[CR45] Tonsing KN (2018). A review of mental health literacy in Singapore. Social Work in Health Care.

[CR46] Tse S, Ng CSM, Yuen WWY, Lo IWK, Fukui S, Goscha RJ, Wan E, Wong S, Chan SK (2021). Process research: Compare and contrast the recovery-orientated strengths model of case management and usual community mental health care. BMC Psychiatry.

[CR47] Tsoi EW, Tse S, Canda ER, Goscha RJ, Lo IW (2022). The meaning of strengths for strengths-based mental health practice in Hong Kong chinese culture: A qualitative exploratory study. Psychiatric Rehabilitation Journal.

[CR48] Waller S, Reupert A, Ward B, McCormick F, Kidd S (2019). Family-focused recovery: Perspectives from individuals with a mental illness. International Journal of Mental Health Nursing.

[CR49] Wyder M, Bland R (2014). The Recovery Framework as a way of understanding families’ responses to Mental illness: Balancing different needs and recovery journeys. Australian Social Work.

[CR50] Yoder HN, de Jong JT, Tol WA, Duncan JA, Bayoh A, Reis R (2021). Child witchcraft confessions as an idiom of distress in Sierra Leone; results of a rapid qualitative inquiry and recommendations for mental health interventions. Child and Adolescent Psychiatry and Mental Health.

[CR51] Yu BCL, Mak WWS, Chio FHN (2021). Family involvement moderates the relationship between perceived recovery orientation of services and personal narratives among chinese with schizophrenia in Hong Kong: A 1-year longitudinal investigation. Social Psychiatry and Psychiatric Epidemiology.

[CR52] Yuan Q, Abdin E, Picco L, Vaingankar JA, Shahwan S, Jeyagurunathan A, Sagayadevan V, Shafie S, Tay J, Chong SA, Subramaniam M (2016). Attitudes to Mental Illness and its demographic correlates among General Population in Singapore. PLoS One.

[CR53] Yuen WW, Tse S, Murray G, Davidson L (2019). From my point of view, my wife has recovered’: A qualitative investigation of caregivers’ perceptions of recovery and peer support services for people with bipolar disorder in a chinese community. International Journal of Social Psychiatry.

[CR54] Yung JYK, Wong V, Ho GWK, Molassiotis A (2021). Understanding the experiences of hikikomori through the lens of the CHIME framework: Connectedness, hope and optimism, identity, meaning in life, and empowerment; systematic review. BMC Psychol.

